# Pilot study identifying distinct circulating proteomic profiles associated with longitudinal CT-defined fibrotic and inflammatory sarcoidosis

**DOI:** 10.3389/fimmu.2026.1888839

**Published:** 2026-08-17

**Authors:** Vibha Shastry, Sonia M. Leach, Brett M. Elicker, Laura L. Koth

**Affiliations:** 1 Department of Medicine, University of California, San Francisco, San Francisco, CA, United States; 2 Department of Microbiology and Immunology, Geisel School of Medicine, Dartmouth College, Lebanon, NH, United States; 3 Department of Radiology & Biomedical Imaging, University of California, San Francisco, San Francisco, CA, United States

**Keywords:** interstitial lung diseases, proteomics, radiology, sarcoidosis, X-rays

## Abstract

**Introduction:**

Pulmonary sarcoidosis exhibits heterogeneous clinical trajectories ranging from self-limited disease resolution to chronic progressive fibrosis, yet reliable biomarkers capable of distinguishing these disease patterns remain lacking. Whether longitudinal CT-defined sarcoidosis phenotypes are associated with distinct circulating molecular signatures remains unknown.

**Methods:**

We performed high-throughput plasma proteomics (SomaScan 11K) in participants with pulmonary sarcoidosis classified into longitudinal chest CT-defined progressive fibrosis, progressive nodular inflammatory disease, or resolving disease trajectories, along with healthy controls. CT phenotypes were assigned based on predefined longitudinal changes in reticulation, traction bronchiectasis, nodular involvement, and mediastinal lymphadenopathy across serial CT scans. One plasma sample per participant was selected from the study visit corresponding to the CT time point at which criteria for the assigned longitudinal phenotype were met. Principal component analysis, hierarchical clustering, pathway enrichment, and correlation-based analyses linking protein expression to quantitative CT features were used to evaluate whether distinct longitudinal CT phenotypes were associated with divergent proteomic signatures.

**Results:**

Principal component analysis and hierarchical clustering suggested partial segregation by CT-defined phenotype. Longitudinal CT phenotypes were associated with distinct pathway-level proteomic signatures, with progressive fibrosis enriched for epithelial–mesenchymal transition signaling, and progressive nodular inflammatory disease enriched for mTORC1, MYC, oxidative phosphorylation, adipogenesis, and fatty acid metabolism pathways. Correlation analyses showed coordinated protein-expression patterns associated with fibrotic CT features and mediastinal lymph node enlargement.

**Discussion:**

These findings suggest that longitudinal CT-defined fibrotic and inflammatory sarcoidosis phenotypes are associated with distinct pathway-level proteomic signatures. This pilot study provides preliminary proof-of-concept evidence that integrating longitudinal CT imaging phenotypes with plasma proteomics may serve as a framework for future mechanistic studies and biomarker discovery in pulmonary sarcoidosis.

## Introduction

Sarcoidosis is a multi-organ, immune-mediated granulomatous disease of unknown etiology characterized by a highly heterogeneous clinical course ranging from self-limited disease resolution to chronic progressive disease associated with organ dysfunction and fibrosis (1–6). This variability complicates clinical management and therapeutic decision-making, as there are currently no reliable biomarkers to distinguish patients at risk for progressive fibrosis from those with predominantly inflammatory or self-limited disease (7).

The biological mechanisms underlying divergent clinical trajectories in sarcoidosis, particularly progression to fibrosis, remain incompletely understood. Radiographic imaging, particularly chest computed tomography (CT), provides a direct assessment of lung involvement and can be used longitudinally to distinguish fibrotic remodeling from inflammatory nodular disease (8–10). Leveraging longitudinal CT imaging to define clinically meaningful disease trajectories offers an opportunity to link molecular profiles with distinct patterns of fibrotic and inflammatory lung disease (9).

Proteomic profiling enables comprehensive assessment of circulating proteins, providing a systems-level readout of biological processes, including immune activation, metabolic signaling, and tissue remodeling (11, 12). In contrast to genomic or transcriptomic approaches, plasma proteomics captures downstream functional outputs of disease biology and may reflect contributions from multiple tissue compartments (13–15). While prior studies have explored circulating biomarkers and proteomic signatures in sarcoidosis, integration of these approaches with longitudinal imaging-defined disease trajectories has been limited, particularly for distinguishing progressive fibrotic disease from predominantly inflammatory or resolving phenotypes (7, 14, 16–22). To our knowledge, this represents one of the first applications of high-dimensional plasma proteomics to a longitudinally CT-phenotyped sarcoidosis cohort and the largest proteomic study focused on imaging-defined fibrotic sarcoidosis phenotypes.

Given the marked clinical heterogeneity of sarcoidosis, approaches that integrate molecular profiling with longitudinal imaging phenotypes may provide insight into biologic processes associated with divergent disease trajectories. In particular, integrating circulating proteomic signals with imaging-derived measures of fibrosis and inflammation may help characterize molecular pathways associated with distinct imaging-defined phenotypes. We hypothesized that longitudinal CT-defined sarcoidosis phenotypes would be associated with distinct circulating proteomic signatures when plasma sampling was anchored to the corresponding imaging-defined trajectory.

In this study, we applied high-throughput plasma proteomics to a cohort of individuals with pulmonary sarcoidosis classified into longitudinal CT-defined phenotypes, including progressive fibrotic disease, progressive nodular inflammatory disease, and resolving disease, along with healthy controls. Plasma samples were selected from study visits corresponding to the CT time point at which predefined imaging criteria for the assigned longitudinal phenotypes were met. As a proof-of-concept investigation, we integrated unsupervised analyses, pathway enrichment, and protein–imaging correlation approaches to determine whether longitudinal CT-defined sarcoidosis phenotypes were associated with biologically coherent circulating proteomic signatures and pathway-level molecular programs. This integrative approach may help define molecular pathways associated with distinct imaging-defined disease phenotypes and identify candidate biomarkers for future validation across fibrotic and non-fibrotic disease trajectories.

## Materials and methods

### Cohort description and CT analysis

This study was performed within a longitudinal cohort of individuals with pulmonary sarcoidosis, as previously described (23). Participants meeting ATS/ERS/WASOG diagnostic criteria (3) and healthy controls without known inflammatory, pulmonary, or systemic disease were recruited from the San Francisco Bay Area between January 1, 2010 and December 31, 2021, with follow-up visits at 6–12-month intervals. CT scans were obtained as part of routine clinical care using standard multidetector chest CT protocols. All participants provided written informed consent, and the study was approved by the UCSF Institutional Review Board in accordance with the Declaration of Helsinki.

Serial chest CT scans obtained at baseline and during follow-up were visually scored by an expert thoracic radiologist blinded to clinical data. Reticular abnormality and nodular disease extent were quantified to the nearest 5% across three zones per lung as previously described (24). Traction bronchiectasis severity was assessed using a standardized semiquantitative approach. Within each of the five lobes, severity was graded by comparing the diameter of the airway to that of the adjacent pulmonary artery using a 4-point scale (0–3): 0, no traction bronchiectasis; 1, mild (airway diameter comparable to the artery); 2, moderate (up to twice the diameter of the artery); and 3, severe (greater than twice the diameter of the artery). Lobar scores were summed to yield a total score ranging from 0 to 15 (25). Mediastinal lymph node size was measured at standard stations (paratracheal, subcarinal, and aortopulmonary window) using the short-axis diameter in millimeters, with ≤10 mm considered within normal limits.

### Trajectory-based CT phenotyping

A nested analysis was performed within the longitudinal cohort. Participants with at least two serial CT scans during follow-up were eligible for CT trajectory phenotyping. Serial semiquantitative CT scores were evaluated longitudinally to identify progressive fibrotic (reticulation, traction bronchiectasis) and nodular imaging changes. Progressive change was defined between baseline and final CT assessment as an increase of ≥5% in reticulation or nodular extent, or an increase of ≥1 unit in traction bronchiectasis score. Participants with stable imaging findings over time were not assigned to a phenotype group because the study focused on predefined progressive and resolving longitudinal CT trajectories.

Participants were classified into three CT trajectory groups based on longitudinal imaging patterns (1): progressive fibrosis, defined by increasing reticulation and/or worsening traction bronchiectasis over time, with or without nodular inflammation (2); progressive nodular disease, defined by increasing nodular involvement without evidence of progressive fibrotic change; and (3) disease resolution, defined by normalization of previously enlarged mediastinal lymph nodes and improvement or resolution of nodular CT abnormalities without progressive fibrotic change.

The analytic cohort consisted of participants meeting criteria for one of these CT trajectory groups, along with healthy controls. One plasma sample per participant was analyzed. Samples were selected from the study visit corresponding to the CT scan at which the participant fulfilled criteria for the assigned CT phenotype. Healthy controls did not undergo CT imaging. Immunosuppressive therapy use at the time of plasma sampling was recorded and incorporated as a sample-level annotation in unsupervised clustering analyses.

### SomaLogic proteomics assay

The SomaScan 11K assay (SomaLogic, Inc., Boulder, CO, USA) quantified >10,000 human protein analytes from previously unthawed archived plasma samples stored at −30 °C. The SOMAmer-based platform uses modified DNA aptamers for hybridization-based fluorescence detection, with protein abundance reported as relative fluorescent units (RFU).

Samples were thawed once and processed in a single SomaScan assay run to minimize batch effects. RFU values were normalized using SomaLogic’s standard pipeline, including hybridization control normalization, intraplate median normalization, plate scaling, calibration, and adaptive normalization.

### Proteomic data processing and filtering

RFU values were log10-transformed for statistical analyses according to SomaScan recommendations unless otherwise specified. For volcano plot visualization, fold change was calculated separately as the log2 ratio of the median normalized RFU values between the progressive fibrosis and progressive nodular disease groups to facilitate conventional interpretation of fold-change magnitude. For principal component analysis and clustering, log10-transformed values were centered and scaled. Signal-to-noise (STN) ratios were calculated as median RFU divided by the assay-specific limit of detection estimated using the calc_eLOD function in the SomaDataIO package in R. Analytes below the empirically determined STN threshold were excluded from selected downstream analyses. No missing analyte values were present following SomaLogic normalization; therefore, no imputation was performed.

### Statistical analyses

Statistical and graphical analyses used R version 4.5.0 and Stata v19 (StataCorp, College Station, TX, USA). Continuous variables were summarized using means and standard deviations, and categorical variables using counts and percentages where group differences were assessed using Kruskal–Wallis tests for continuous variables and Fisher’s exact tests for categorical variables. Principal component analysis (PCA) was performed using the prcomp function from the R stats package on log10-transformed, standardized RFU values. Sample-level outliers were evaluated using PCA and median absolute deviation (MAD) analysis (26). Following SomaLogic guidance, samples exceeding 6 MAD from the median were excluded. Global protein expression patterns were evaluated by unsupervised hierarchical clustering of log10-transformed, standardized RFU values from analytes passing STN filtering. Samples and proteins were clustered using correlation-based distance (1 − Pearson correlation) and Ward’s minimum variance (Ward D2) linkage and visualized using the pheatmap R package. Differential expression between progressive fibrosis and progressive nodular disease phenotypes was assessed using analyte-wise Welch two-sample t-tests on log10-transformed, centered, and scaled RFU values, following the standard SomaLogic analytical workflow for two-group comparisons. False discovery rate (FDR) was controlled using the Benjamini–Hochberg procedure, with FDR < 0.05 considered statistically significant. Exploratory sensitivity analyses were performed for proteins meeting the predefined FDR < 0.05 threshold using linear regression models with sequential adjustment for sex, immunosuppressive therapy at the time of plasma sampling, and both covariates simultaneously. Gene set enrichment analysis (GSEA) was performed using the pre-ranked method implemented in the R *fgsea* package with the Hallmark gene set collection from MSigDB. Protein analytes were mapped to gene identifiers and ranked using a signed significance metric, calculated as sign(log2 fold change) × −log10(p-value), where log2 fold change was calculated as the log2 ratio of the median normalized RFU values in the progressive fibrosis and progressive nodular disease groups. Enrichment analyses were restricted to analytes measured by the SomaScan platform to ensure that pathway enrichment was evaluated relative to the measured proteomic background. For correlation-based analyses, proteins were selected based on differential expression results at a relaxed false discovery rate threshold (FDR ≤ 0.06). Spearman correlation coefficients were calculated between protein expression levels and CT imaging features or biomarker measurements. Correlation matrices were visualized as clustered heatmaps, with color intensity representing the magnitude and direction of correlation. Hierarchical clustering was applied to protein analytes and the corresponding CT imaging features or blood biomarkers using correlation-based distance (1 − Spearman correlation) and average linkage. Heatmaps were generated using the ComplexHeatmap R package.

## Results

### Cohort and CT phenotype characteristics

Baseline demographic and clinical characteristics across CT phenotype groups are summarized in **Table 1**, including age, sex, race, smoking history, body mass index, and use of immunosuppressive therapy at the time of plasma sampling. Routine laboratory markers, including C-reactive protein, erythrocyte sedimentation rate, angiotensin-converting enzyme levels, and absolute lymphocyte counts, are summarized in **Table 2**. Absolute lymphocyte counts were numerically lower in the progressive fibrosis group. Clinical features and pulmonary function measurements at the time of plasma sampling are summarized in **Table 3**. Mean lung function parameters were lowest in the progressive fibrosis group, particularly for FEV1/FVC and FEV1 measures.

**Table 1:** Clinical characteristics at date of plasma sampling*.

	CT Phenotype			
	Healthy control	Resolved CT features	Progressive nodular	Progressive fibrosis
N	5	5	15	15
Age (yr)	52 (4.4)	47 (8.9)	49 (12.1)	53 (10.1)
Sex (count, %)				
Male	2 (40%)	0 (0%)	10 (67%)	6 (40%)
Female	3 (60%)	5 (100%)	5 (33%)	9 (60%)
Race (count, %)				
White	3 (60%)	3 (60%)	12 (80%)	10 (67%)
Black	2 (40%)	1 (20%)	1 (7%)	2 (13%)
Other	0 (0%)	1 (20%)	2 (13%)	3 (20%)
Latino (count, %)				
Yes	0 (0%)	0 (0%)	3 (20%)	1 (7%)
Smoking history (count, %)				
Yes	2 (40%)	1 (20%)	7 (47%)	8 (53%)
Pack-year Smoking History	0.2 (0.3)	3 (6.7)	3 (6.5)	3 (4.2)
BMI	29 (5.0)	30 (6.3)	30 (6.5)	26 (2.9)
Taking immunosuppression on blood draw date (count, %)				
Yes	0 (0%)	0 (0%)	3 (20%)	5 (33%)

*all values presented as mean ± SD unless otherwise indicated; data are presented descriptively; formal statistical comparisons were not performed.

**Table 2:** Clinical laboratory blood test results at date of plasma sampling*.

	CT Phenotype			
	Healthy control	Resolved CT features	Progressive nodular	Progressive fibrosis
N	5	5	15	15
Sedimentation Rate (ESR) (mm/h)	9.6 (6.3)	10.3 (5.4)	9.7 (12.4)	13.8 (16.8)
C-Reactive Protein (mg/L)	3.8 (2.4)	4.1 (2.9)	4.1 (2.5)	11.1 (21.9)
Angiotensin Converting Enzyme (U/L)	41 (12.5)	28 (2.8)	80 (69.5)	54 (34.9)
Absolute Lymphocytes (x10^9^/L)	1.96 (0.4)	1.73 (0.7)	1.28 (0.5)	1.11 (0.5)

*all values presented as mean ± SD; data are presented descriptively; formal statistical comparisons were not performed.

**Table 3:** Clinical features at date of plasma sampling*.

	CT Phenotype			
	Resolved CT features	Progressive nodular	Progressive fibrosis	P-value
N	5	15	15	
4 or more organs involved (count, %)				
Yes	3 (60%)	6 (40%)	4 (27%)	0.401
FEV1/FVC	0.79 (0.06)	0.74 (0.08)	0.68 (0.07)	0.031
FEV1 z-score	0.347 (0.5)	-0.395 (1.3)	-1.183 (1.0)	0.026
FEV1% predicted	105 (7.8)	95 (19.9)	80 (18.1)	0.020
FVC z-score	0.533 (0.7)	0.168 (1.3)	-0.396 (1.0)	0.262
FVC% predicted	108 (10.1)	103 (19.7)	93 (16.9)	0.221
DLCO z-score	-0.416 (1.552)	-1.552 (1.276)	-1.662 (2.177)	0.604
DLCO% predicted	95 (21.6)	80 (14.9)	79 (25.3)	0.547

*all values presented as mean ± SD unless otherwise indicated. P-values were calculated using Fisher’s exact test for categorical variables and Kruskal–Wallis tests for continuous variables.

Chest CT features at the time of plasma sampling differed across trajectory-defined phenotype groups (**Table 4**). The resolved group demonstrated minimal or no evidence of reticulation or traction bronchiectasis. The progressive nodular group was characterized by the greatest extent of nodular involvement and larger mediastinal lymph nodes, whereas the progressive fibrosis group exhibited the highest extent of reticulation and greatest traction bronchiectasis severity. Duration of longitudinal CT follow-up and the interval between biopsy-confirmed diagnosis and plasma sampling demonstrated substantial overlap across phenotype groups (**Table 4**). These imaging differences reflect the predefined trajectory-based phenotype classifications derived from longitudinal changes in reticulation, nodular involvement, and traction bronchiectasis.

**Table 4:** Chest CT scan features at date of plasma sampling*.

	CT Phenotype			
	Resolved CT features	Progressive nodular	Progressive fibrosis	P-value
N	5	15	15	
Percent of Lung Involved with Nodules	4 (6.5)	41 (19.0)	23 (23.8)	0.002
Percent of Lung Involved with Reticulation	0 (0.0)	1 (2.6)	18 (16.4)	<0.001
Traction Bronchiectasis Score (count, %)**				
0	5 (100%)	12 (80%)	1 (7%)	<0.001
2	0 (0%)	2 (13%)	4 (27%)	
3	0 (0%)	1 (7%)	1 (7%)	
4	0 (0%)	0 (0%)	5 (33%)	
8	0 (0%)	0 (0%)	1 (7%)	
9	0 (0%)	0 (0%)	3 (20%)	
Lymph Node Size AP Window (mm)	5 (2.1)	11 (4.0)	7 (2.6)	0.002
Lymph Node Size Paratracheal (mm)	7 (4.2)	13 (5.4)	9 (4.7)	0.041
Lymph Node Size Subcarinal (mm)	8 (4.3)	15 (4.0)	11 (4.9)	0.009
Years between baseline and last follow-up CT scan	4.6 (2.6)	6.3 (4.2)	5.7 (3.9)	0.731
Years between date of biopsy diagnosis and sample time point	4.5 (5.0)	6.3 (4.7)	8.2 (6.8)	0.372

*all values presented as mean ± SD unless otherwise indicated. P-values represent overall comparisons across CT phenotype groups using Kruskal–Wallis tests for continuous variables and Fisher’s exact test for categorical variables.

**Only observed scores are shown.

### Global data overview, quality control, and principal component analysis

The SomaScan assay measured 10,760 plasma protein analytes across 35 participants with sarcoidosis and five healthy controls. Principal component analysis (PCA) identified four samples with outlying global proteomic profiles. Robust outlier analysis based on median absolute deviation independently identified the same four samples. Three of these samples were also flagged by SomaLogic quality control procedures. The excluded samples included three participants from the progressive nodular phenotype group and one participant from the resolved group.

**Figure 1:**
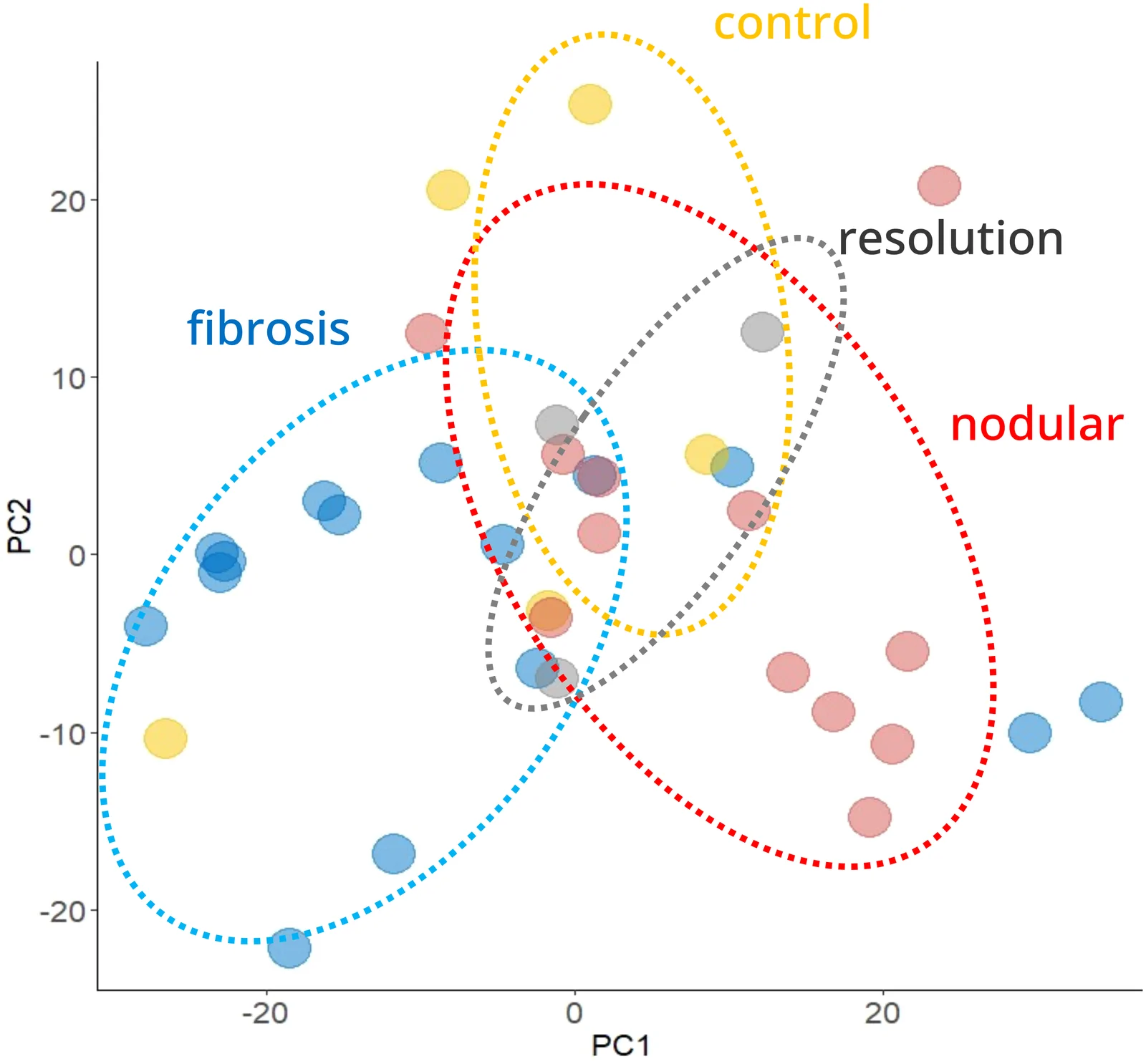
Principal component analysis (PCA) of plasma proteomics profiles by phenotype group. PCA plot of plasma proteomic profiles following exclusion of four samples identified as outliers by principal component analysis and robust outlier analysis. The plot included 2,006 analytes retained following signal-to-noise (STN) filtering based on an inflection point identified at an STN ratio of 31.8. Each point represents an individual sample and is colored according to phenotype group. Ellipses represent phenotype group distributions.

To focus downstream analyses on higher-confidence protein measurements while reducing dimensionality and the burden of multiple testing, analytes were filtered based on the distribution of signal-to-noise (STN) ratios. An inflection point was identified at an STN ratio of 31.8, yielding a subset of approximately 2,000 analytes for downstream analyses (**Supplementary Figure 1**). This approach preferentially retained analytes with greater variance across samples (**Supplementary Figure 2**). PCA performed on the filtered dataset demonstrated partial separation between progressive fibrosis and progressive nodular disease phenotypes (**Figure 1**), with some overlap among groups. Principal component 1 (PC1) explained 27% of the variance and principal component 2 (PC2) explained 12%.

### Exploratory analysis via unsupervised clustering

**Figure 2:**
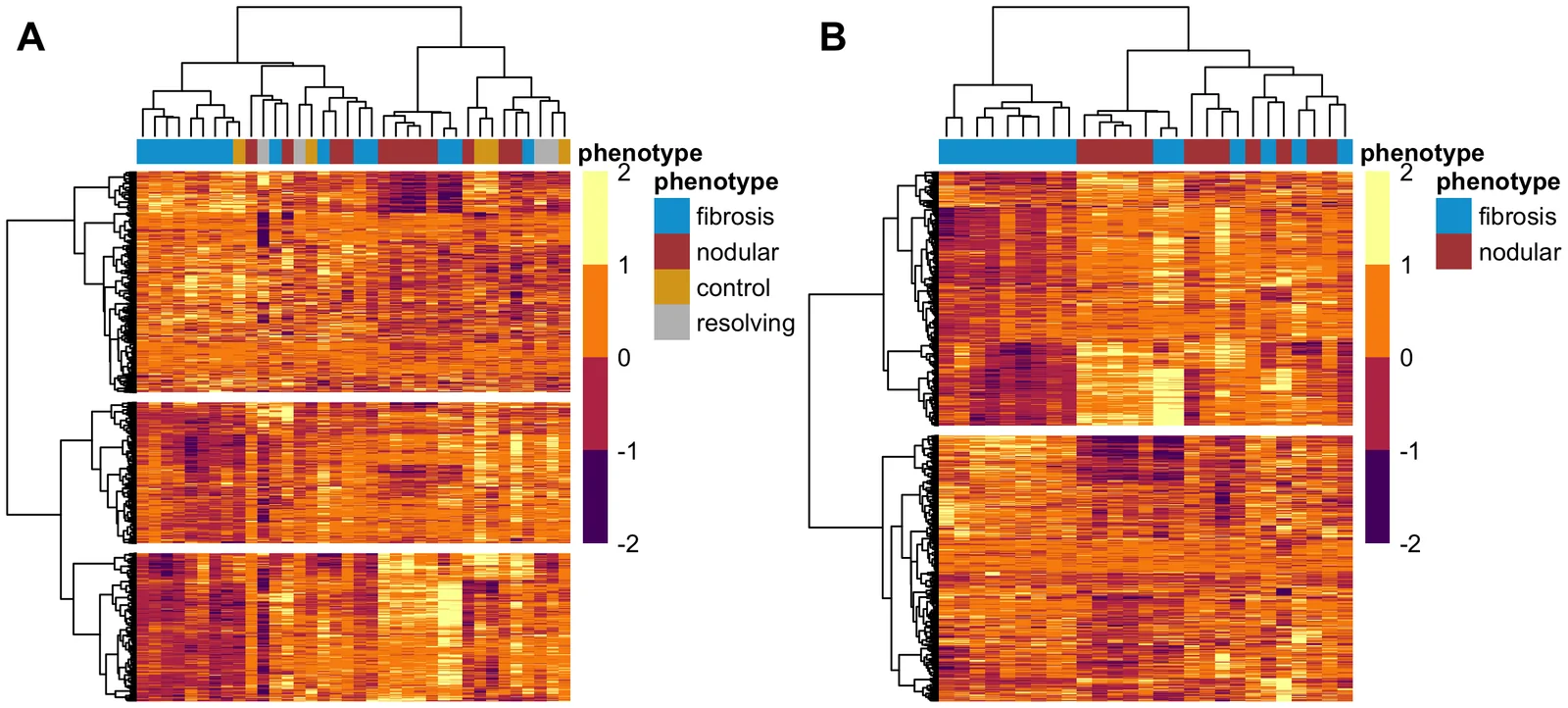
Unsupervised hierarchical clustering of plasma proteomic profiles. Heatmaps were generated using the 2,006 analytes retained following signal-to-noise (STN) filtering based on an inflection point identified at an STN ratio of 31.8. Relative fluorescent unit (RFU) values were log10-transformed, centered, and scaled prior to clustering. Unsupervised hierarchical clustering was performed on both samples and proteins using correlation-based distance (1 − Pearson correlation) and Ward D2 linkage. Rows represent proteins and columns represent individual samples. **(A)** Heatmap including all CT phenotypes and healthy controls. Three major protein clusters were identified, containing 554, 869, and 583 analytes, respectively. **(B)** Heatmap restricted to the progressive fibrosis and nodular disease phenotypes. Two major protein clusters were identified, containing 1,169 and 869 analytes, respectively.

Unsupervised hierarchical clustering was performed to visualize global patterns of protein expression across all phenotype groups using the filtered high-confidence analyte set (**Figure 2A**). Clustering of participants demonstrated broad separation of progressive fibrosis and progressive nodular disease phenotypes, although overlap among groups remained. Clustering of proteins identified three major analyte clusters with distinct expression patterns across phenotype groups, comprising 554, 869, and 583 analytes, respectively.

To further examine differences between progressive fibrosis and progressive nodular disease phenotypes, clustering was repeated using only these two groups (**Figure 2B**). This analysis demonstrated two primary protein clusters, containing 1,169 and 869 analytes. Visual inspection of sample-level annotations did not reveal an obvious clustering pattern according to immunosuppressive therapy use or sex (**Supplementary Figure 3**). The contribution of these variables to the overall clustering patterns across the full proteome was not formally assessed and therefore cannot be excluded.

### Differential expression and enrichment analyses

**Figure 3:**
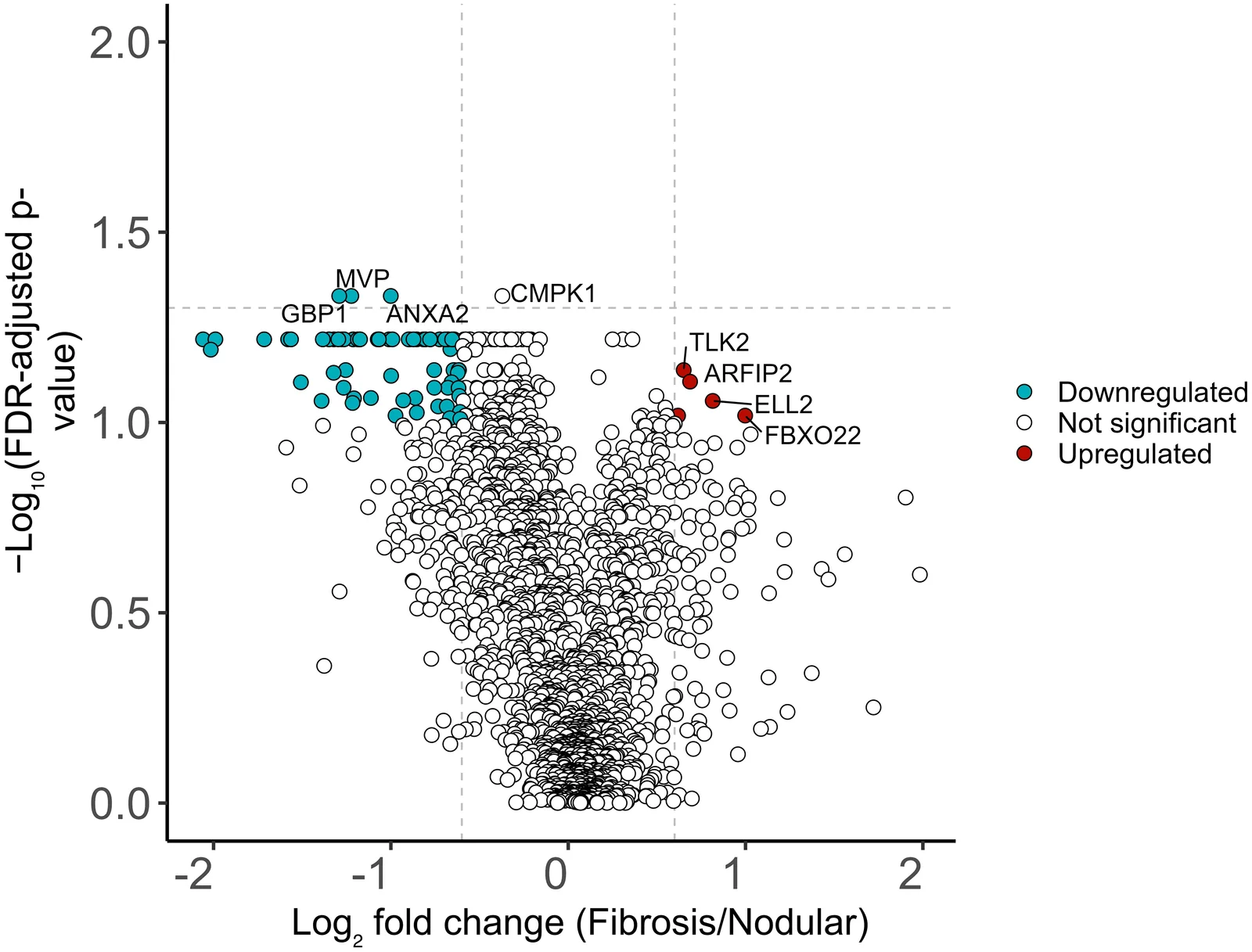
Volcano plot comparing protein analyte expression between the progressive fibrosis and progressive nodular disease CT phenotypes. The analysis included 2,006 analytes passing the predefined signal-to-noise threshold. Fold-change values were calculated as the log2 ratio of the median normalized relative fluorescent unit (RFU) values in the progressive fibrosis and progressive nodular disease groups. Analytes were classified as upregulated (red) or downregulated (teal) in progressive fibrosis using a log2 fold-change threshold of ±0.6 (equivalent to an absolute fold change ≥1.5; vertical dashed lines) and an FDR-adjusted p value threshold of 0.1 (horizontal dashed line). Four downregulated analytes (GBP1, MVP, ANXA2, and CMPK1) met a stricter FDR threshold of 0.05, although CMPK1 did not meet the predefined fold-change threshold.

Differential expression analysis comparing progressive fibrosis and progressive nodular disease phenotypes identified a relatively small number of proteins meeting FDR < 0.05 (**Figure 3**). Exploratory sensitivity analyses were performed for the three proteins meeting the FDR < 0.05 threshold using linear regression models adjusted for sex, immunosuppressive therapy, and both covariates simultaneously. Adjustment produced only modest changes in the phenotype-associated regression coefficients (≤11%), while preserving the direction and statistical significance of all three associations (**Supplementary Table 1**).

**Figure 4:**
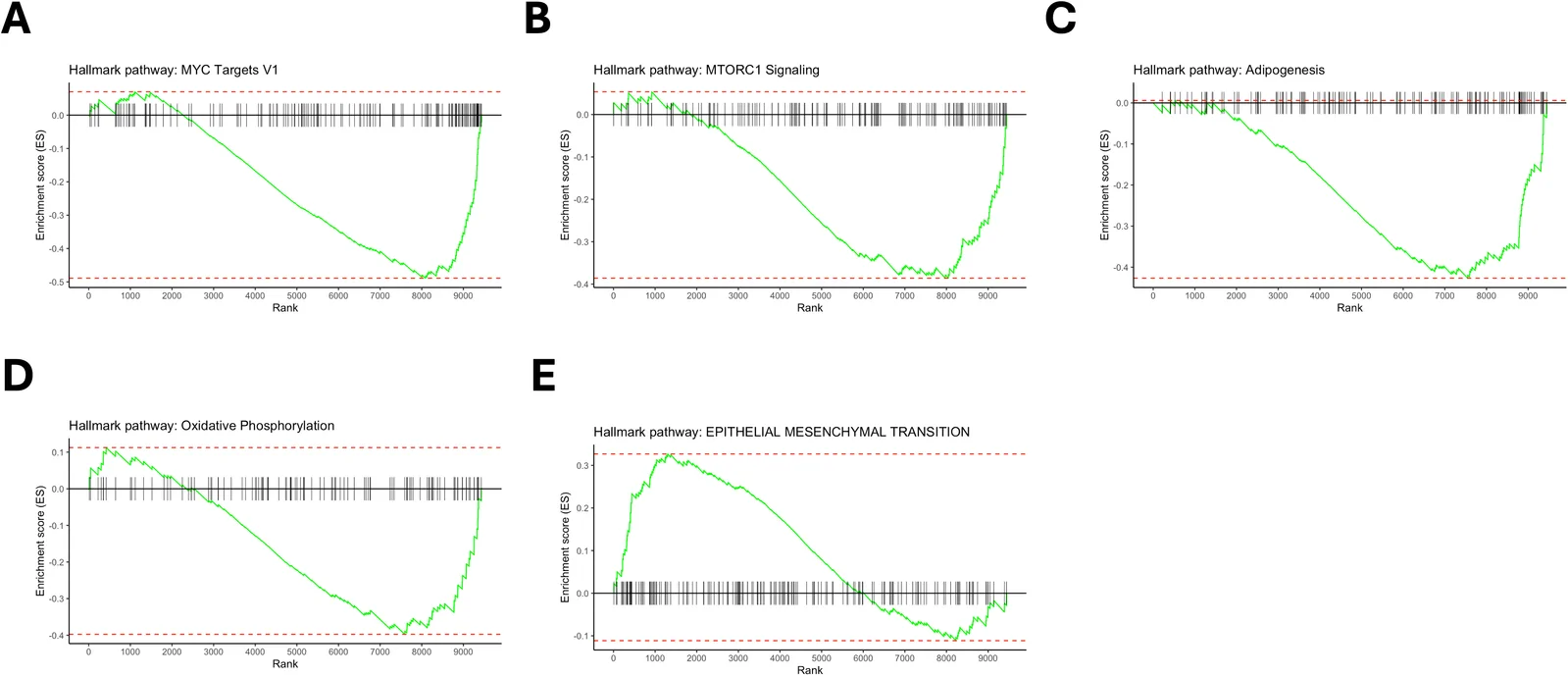
Gene set enrichment analysis (GSEA) of proteins ranked by differential expression between the progressive fibrosis and progressive nodular disease CT phenotypes. Enrichment plots from the Hallmark gene set collection are shown for **(A)** MYC Targets V1, **(B)** MTORC1 Signaling, **(C)** Adipogenesis, **(D)** Oxidative Phosphorylation, and **(E)** Epithelial Mesenchymal Transition. Gene sets shown in **(A-D)** were enriched in the progressive nodular disease phenotype relative to progressive fibrosis, whereas the Epithelial Mesenchymal Transition gene set **(E)** was enriched in progressive fibrosis relative to the progressive nodular phenotype.

To examine coordinated pathway-level differences in protein expression between these groups, gene set enrichment analysis (GSEA) was performed using a pre-ranked proteomic analyte list, as described in Methods. Hallmark pathways enriched in the progressive nodular phenotype included mTORC1 signaling (normalized enrichment score, NES = -1.6; P_adjusted_ = 0.007), oxidative phosphorylation (NES = -1.5; P_adjusted_ = 0.026), adipogenesis (NES = -1.7; P_adjusted_ = 0.003), fatty acid metabolism (NES = -1.4; P_adjusted_ = 0.026), and MYC signaling (NES = -2.1; P_adjusted_ <0.001) (**Figures 4A–D**). In contrast, the epithelial–mesenchymal transition (EMT) pathway was enriched in the progressive fibrosis phenotype (NES = 1.5; P_adjusted_ = 0.015) (**Figure 4E**).

### Correlation and heatmap analysis of protein expression with CT features and blood biomarkers

**Figure 5:**
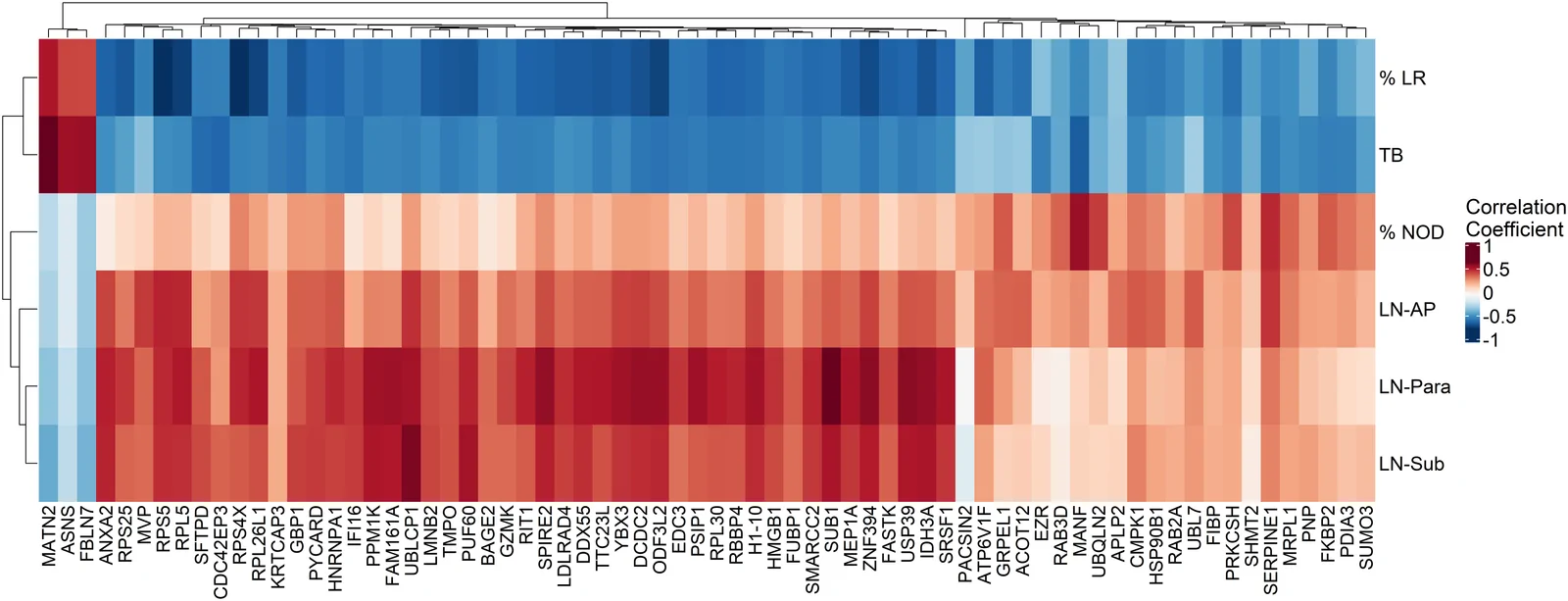
Heatmap of Spearman correlation coefficients between selected protein analytes and chest CT imaging features. The analysis was restricted to participants with progressive fibrosis and progressive nodular disease phenotypes. Protein analytes were selected from the differential expression analysis using a false discovery rate threshold of ≤ 0.06. The color scale represents the strength and direction of Spearman correlation coefficients (red = positive, blue = negative). Unsupervised hierarchical clustering was performed on both protein analytes and CT imaging features using correlation-based distance (1 − Spearman correlation) and average linkage. CT imaging features, listed from top to bottom, include percentage of lung reticulation (%LR), severity of traction bronchiectasis (TB), percentage of nodular opacities (%NOD), and the size of the largest mediastinal lymph node in the aortopulmonary window (LN-AP), paratracheal (LN-Para), and subcarinal (LN-Sub) stations.

To evaluate relationships between protein expression and imaging-derived disease features, Spearman correlation coefficients were calculated between chest CT features and analytes selected from differential expression analysis using a relaxed false discovery rate threshold (FDR ≤ 0.06) (**Figure 5**). Protein analyte expression demonstrated distinct correlation patterns across CT-derived imaging features. Correlations with fibrotic features, including reticulation and traction bronchiectasis, differed in both direction and magnitude from those associated with nodular involvement. Lymph node size showed moderate positive correlations with a subset of proteins.

**Figure 6:**
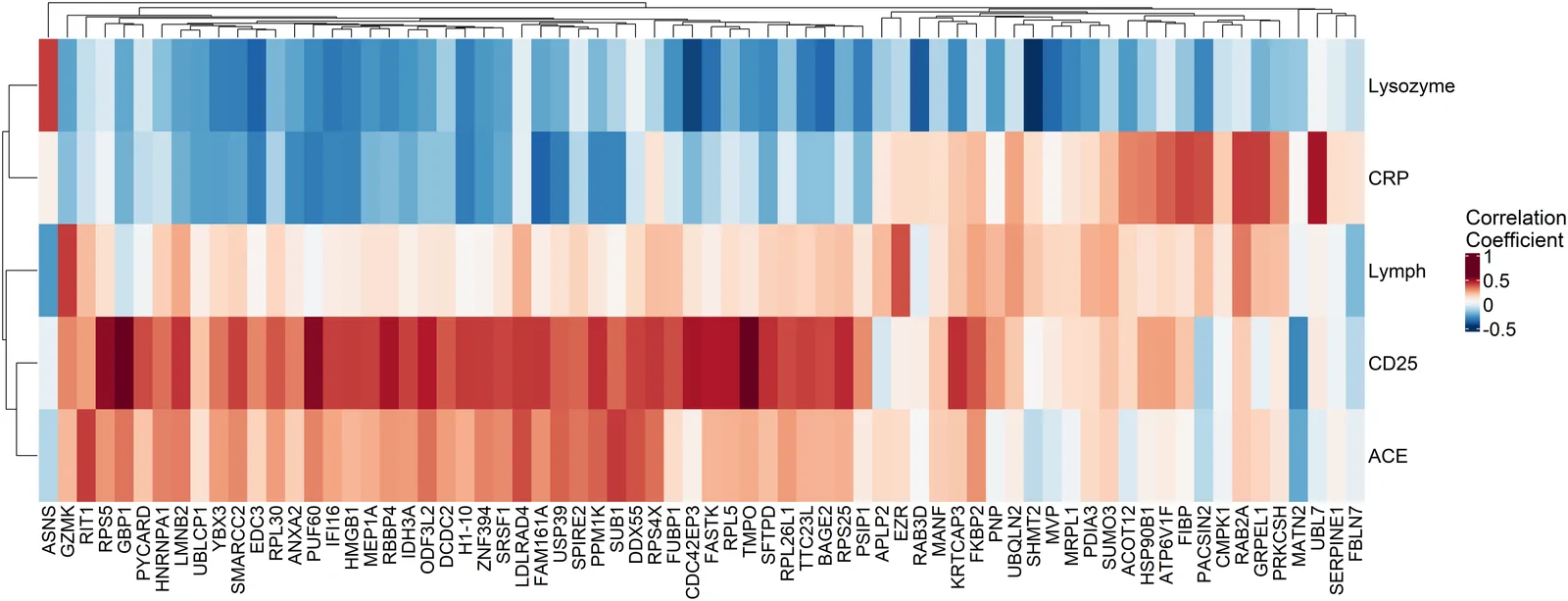
Heatmap of Spearman correlation coefficients between selected protein analytes and blood biomarkers. The analysis was restricted to participants with progressive fibrosis and progressive nodular disease phenotypes. Protein analytes were selected from the differential expression analysis using a false discovery rate threshold of ≤ 0.06. The color scale represents the strength and direction of Spearman correlation coefficients (red = positive, blue = negative). Unsupervised hierarchical clustering was performed on both protein analytes and blood biomarkers using correlation-based distance (1 − Spearman correlation) and average linkage. The blood biomarkers, listed from top to bottom, include lysozyme (ng/mL), C-reactive protein (mg/L) (CRP), absolute lymphocyte count (10^9^/L) (Lymph), soluble IL-2Ra (pg/mL) (CD25), and angiotensin-converting enzyme (U/L) (ACE).

Correlations between protein expression and circulating clinical biomarkers were also assessed (**Figure 6**). Clinical biomarkers including soluble IL-2 receptor, angiotensin-converting enzyme, and lymphocyte count demonstrated predominantly weak correlations with protein expression. In contrast, C-reactive protein and lysozyme demonstrated weak to moderate negative correlations with most proteins, although a subset of proteins showed positive associations with C-reactive protein.

## Discussion

This pilot study supports the potential value of integrating longitudinal high-resolution chest CT phenotyping with deep plasma proteomics in sarcoidosis. In this rigorously characterized pilot cohort, pathway-level differences were observed between CT-defined sarcoidosis phenotypes, with enrichment of metabolic pathways in progressive nodular disease and epithelial-mesenchymal transition (EMT)-associated pathways in progressive fibrosis.

Although relatively few individual proteins met the predefined false discovery rate threshold, several, including ANXA2, GBP1, and MVP, have previously been implicated in fibrotic lung disease or cellular stress responses (27–30). Several have also been reported in idiopathic pulmonary fibrosis, suggesting overlap between the proteomic features identified here and pathways involved in established fibrotic lung disease (31, 32). However, given the limited sample size and modest differential expression signal, these findings should be considered hypothesis-generating and require independent validation.

The pathway analysis suggested enrichment of molecular programs associated with the progressive nodular disease phenotype. Enrichment of mTORC1 signaling, oxidative phosphorylation, adipogenesis, fatty acid metabolism, and MYC signaling suggests that coordinated metabolic reprogramming may contribute to the biology of progressive inflammatory sarcoidosis. The mTORC1 finding aligns with experimental evidence that chronic macrophage mTORC1 activation promotes granuloma formation and proinflammatory remodeling, providing a potential mechanistic link to persistent granulomatous inflammation (33). MYC signaling, which regulates cell proliferation and growth, has also been associated with multinucleated macrophage subsets involved in granuloma development (34).

Pathway analyses in the progressive fibrosis phenotype were consistent with activation of tissue remodeling pathways, including epithelial–mesenchymal transition (EMT), a program implicated in fibrotic remodeling and extracellular matrix production in chronic lung disease (35–37). Although the modest cohort limited individual protein-level differential expression, several analytes identified in exploratory analyses have previously been linked to extracellular matrix remodeling and fibrosis. These included plasminogen activator inhibitor-1 (PAI-1), a regulator of TGF-β-associated profibrotic signaling (38–40), and the extracellular matrix-associated proteins Matrilin-2 (41, 42) and Fibulin-7 (42), which have been implicated in tissue remodeling and fibroblast activation. Collectively, these findings are consistent with the biologic relevance of the imaging-defined progressive fibrosis phenotype and suggest shared molecular programs with other fibrotic lung diseases. These observations should therefore be interpreted as supportive of pathway-level activity rather than evidence of specific mechanistic drivers.

A major strength of this study is the availability of longitudinally collected plasma samples spanning multiple years, allowing proteomic profiling to be aligned with the time points at which CT-defined disease trajectories were established. Integrating longitudinal CT-defined phenotyping with plasma proteomics anchored molecular analyses to clinically meaningful disease evolution rather than cross-sectional disease status alone. Because proteomics captures protein-level biological activity, including downstream signaling and post-translational processes, this approach identified candidate molecular signatures and pathway-level associations with imaging-defined disease manifestations.

Several limitations should be considered. The modest sample size limits generalizability and reduced statistical power for detecting differences at the individual protein level. Although sex distribution differed across phenotype groups, exploratory sensitivity analyses adjusting for sex and immunosuppressive therapy produced only modest changes in the phenotype-associated effect estimates for the three proteins meeting the predefined FDR threshold. Nevertheless, the modest cohort size precludes excluding residual confounding or performing reliable covariate-adjusted analyses across the full proteome. The study was designed as a cross-sectional proteomic analysis anchored to longitudinal CT-defined phenotypes and was not intended to evaluate prediction of future fibrosis, clinical progression, treatment response, or physiologic decline. In addition, the focus on progressive and resolving CT-defined phenotypes excluded participants with stable imaging findings; therefore, the extent to which stable disease may be associated with distinct proteomic signatures remains unknown. Finally, the absence of an independent validation cohort limits assessment of the reproducibility and external generalizability of these findings. Despite these limitations, the concordance between the observed pathway-level signatures and established mechanisms of granulomatous inflammation and fibrosis supports further validation of these findings in larger independent cohorts.

In summary, this pilot study supports the potential value of quantitative longitudinal CT phenotyping combined with plasma proteomics to investigate biological heterogeneity in sarcoidosis. The distinct pathway-level signatures observed in this study warrant further investigation and validation in larger independent cohorts.

## Data Availability

De-identified data supporting the findings of this study are available from the corresponding author upon reasonable request and with appropriate institutional approval.
