# Supplementary material for: Pilot study identifying distinct circulating proteomic profiles associated with longitudinal CT-defined fibrotic and inflammatory sarcoidosis
Source: Front Immunol. 2026 Aug 17;17:1888839. doi: 10.3389/fimmu.2026.1888839 (PMC13526994; doi:10.3389/fimmu.2026.1888839)
Supplement: Supplementary file 1 [file SupplementaryFile1.pdf]

Supplemental Figures

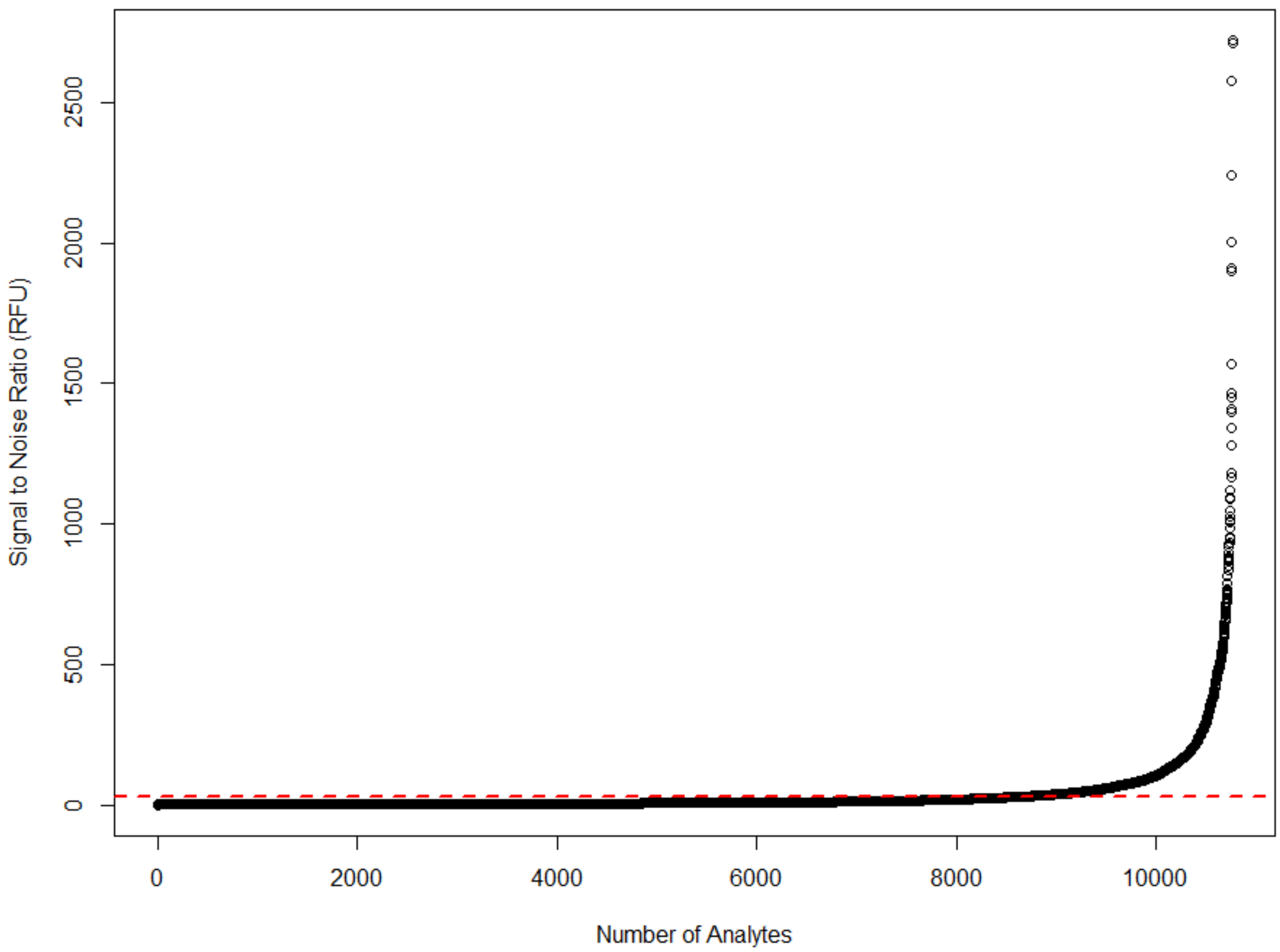

Supplemental Figure 1: Signal-to-noise filtering of SomaScan analytes. Scatterplot of analyte-level signal-to-noise ratio (STN) versus median relative fluorescence units (RFU) across plasma samples. STN was calculated for each analyte as the ratio of median RFU to the assay-specific limit of detection, estimated using the calc_eLOD function of the SomaDataIO package in R, consistent with standard SomaScan analytical workflow. The curve demonstrated an empirical inflection point at an STN ratio of 31.8. Applying this threshold retained approximately 2,000 analytes for downstream analyses.

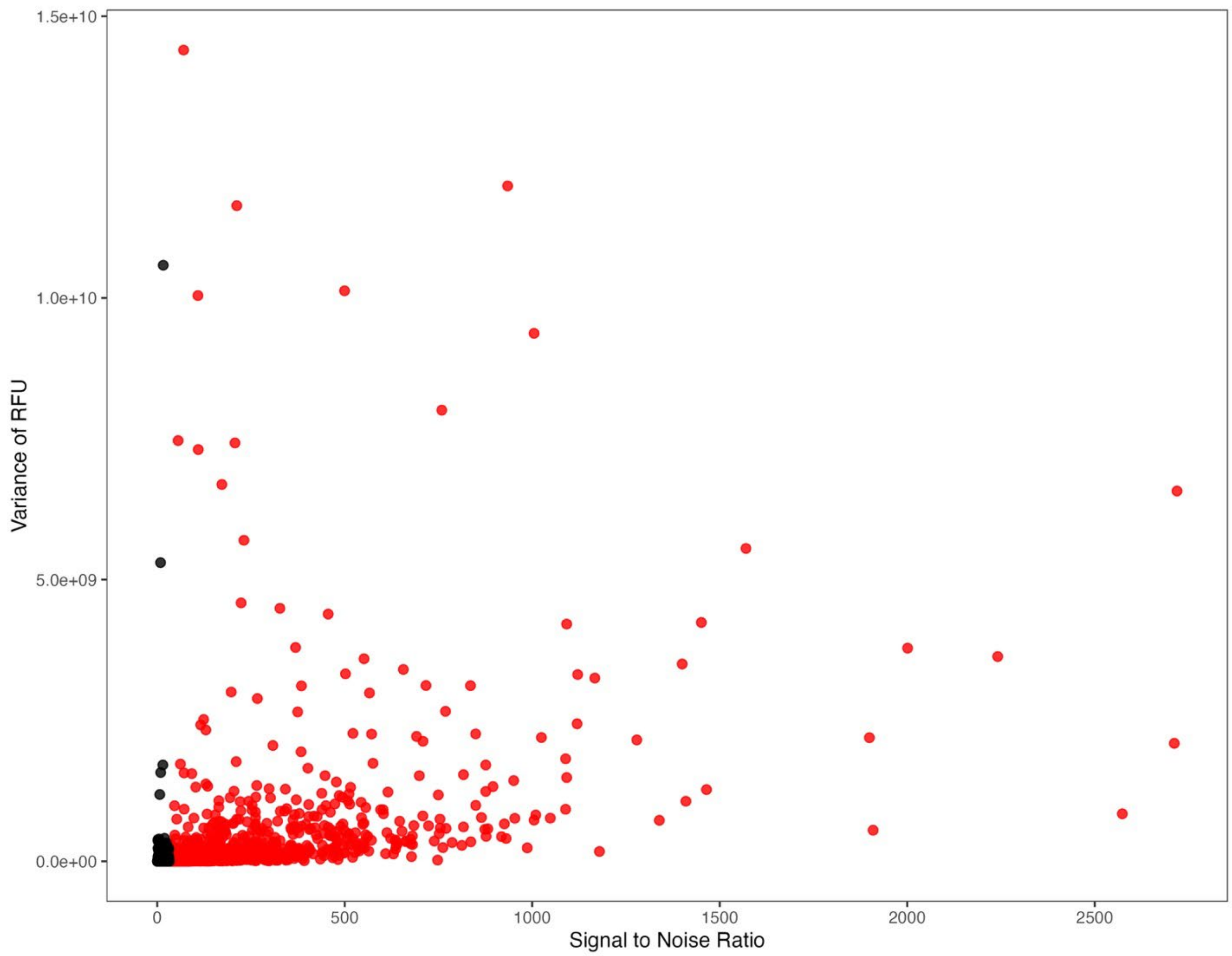

Supplemental Figure 2: **Relationship between analyte variance and signal-to-noise ratio.** Scatterplot showing analyte-level variance plotted against signal-to-noise ratio (STN). Each point represents one SomaScan analyte. Variance was calculated using relative fluorescence unit (RFU) values prior to log transformation and scaling using the full dataset before exclusion of quality-control outlier samples. Red points indicate the approximately 2,000 analytes exceeding the STN threshold of 31.8 identified in Supplemental Figure 1. Analytes exceeding the STN threshold generally demonstrated greater variance across samples, supporting retention of higher-information analytes for downstream analyses.

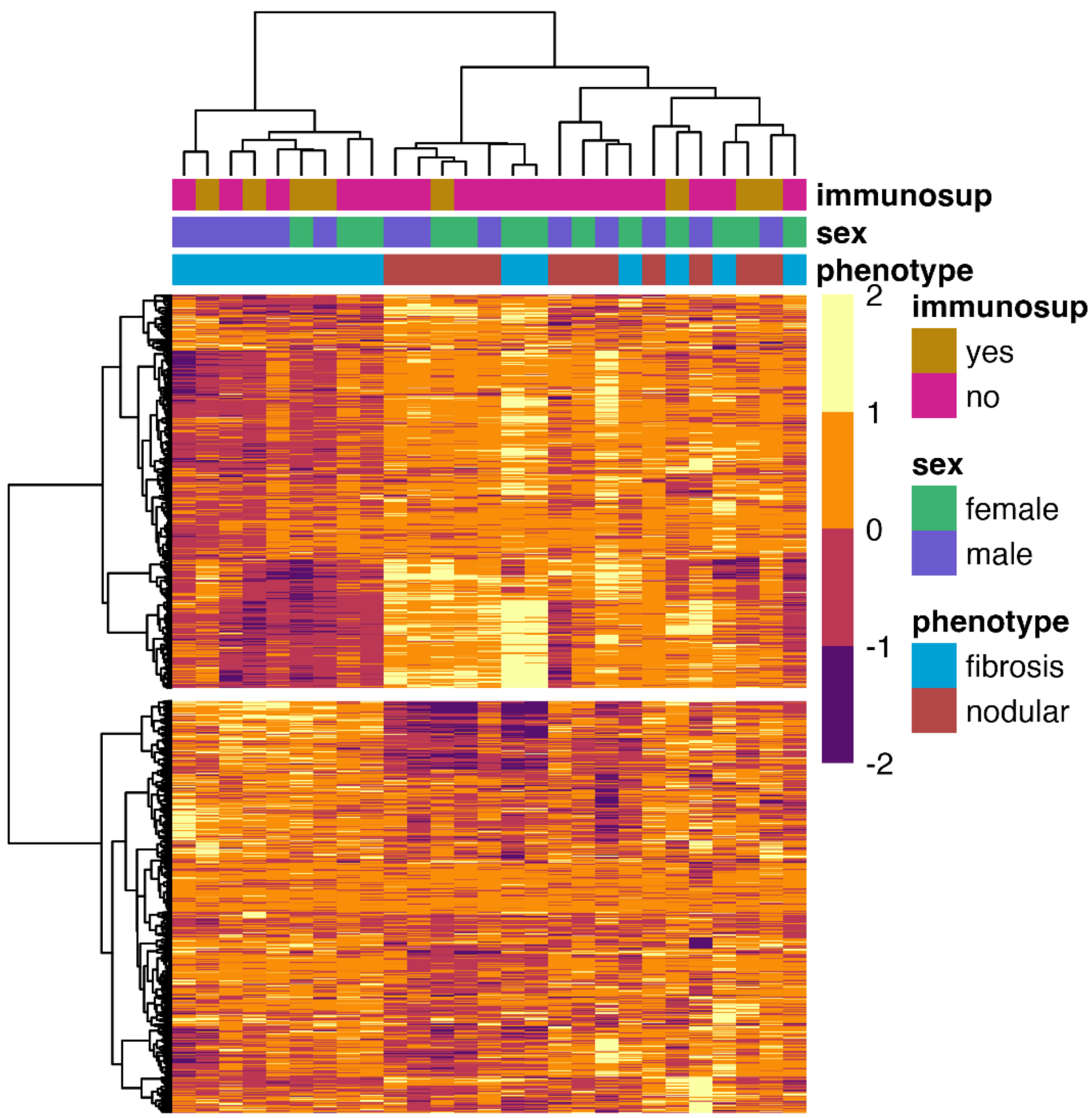

Supplemental Figure 3: Unsupervised hierarchical clustering with sample-level clinical annotations. Heatmap generated using the 2,006 analytes retained following signal-to-noise filtering in participants with progressive fibrosis and progressive nodular sarcoidosis CT phenotypes. RFU values were log10-transformed, centered, and scaled prior to clustering. Unsupervised hierarchical clustering was performed on both samples and protein analytes using correlation-based distance (1 − Pearson correlation) and Ward D2 linkage. Sample-level annotation bars indicate CT phenotype, sex, and immunosuppressive therapy use at the time of plasma sampling. Formal statistical assessment of the contribution of these variables to clustering patterns was not performed.

Supplemental Table 1*: Exploratory sensitivity analyses of differentially expressed proteins after adjustment for sex and immunosuppressive therapy.

**Protein**	**Unadjusted Mean Difference**	**+ Sex**	**+ IS**	**+ Sex + IS**	**Maximum Percent Change**
ANXA2	-0.271	-0.244	-0.268	-0.240	11
GBP1	-0.352	-0.335	-0.352	-0.335	5
MVP	-0.315	-0.345	-0.304	-0.334	10

* Regression coefficients (β) representing the unadjusted and adjusted difference in log10-transformed protein expression between progressive fibrosis and progressive nodular disease
